# *SlWUS1*; An X-linked Gene Having No Homologous Y-Linked Copy in *Silene latifolia*

**DOI:** 10.1534/g3.112.003749

**Published:** 2012-10-01

**Authors:** Yusuke Kazama, Kiyoshi Nishihara, Roberta Bergero, Makoto T. Fujiwara, Tomoko Abe, Deborah Charlesworth, Shigeyuki Kawano

**Affiliations:** * RIKEN Innovation Center, Wako, Saitama 351-0198, Japan; † RIKEN Nishina Center, Wako, Saitama 351-0198, Japan; ‡ Department of Integrated Biosciences, Graduate School of Frontier Sciences, The University of Tokyo, Kashiwa, Chiba 277-8562, Japan; § Institute of Evolutionary Biology, University of Edinburgh, School of Biological Sciences, Edinburgh EH9 3JT, United Kingdom

**Keywords:** dioecious plant, *Silene latifolia*, X-linkage, genetic degeneration, *WUSCHEL*

## Abstract

The dioecious plant *Silene latifolia* has heteromorphic sex chromosomes, and comparison of the positions of sex-linked genes indicates that at least three large inversions have occurred during the evolution of the Y chromosome. In this article, we describe the isolation of a new sex-linked gene from *S. latifolia*, which provides new information on the evolution of this plant’s young sex chromosomes. By using reverse-transcription polymerase chain reaction degenerate primers based on the *Arabidopsis thaliana* sequence of *WUSCHEL*, a flower-development gene, we found two copies in *S. latifolia*, which we named *SlWUS1* and *SlWUS2*. Southern blot and genetic segregation analysis showed that *SlWUS1* is located on the X chromosome and *SlWUS2* is autosomal. No Y-linked copy of *SlWUS1* was found by either Southern blot analysis under low-stringency conditions or polymerase chain reaction with degenerate primers, so we conclude that *SlWUS1* probably has no Y-linked homolog. It is unknown whether the Y chromosome lost the *SlWUS1* copy by degeneration of this individual gene or whether deletion of a larger genome region was involved. Several tests lead us to conclude that dosage compensation has not evolved for this sex-linked gene. We mapped the ortholog in the nondioecious relative *S. vulgaris* (*SvWUS1*), to compare the location in a species that has no history of having sex chromosomes. *SvWUS1* maps to the same linkage group as other fully X-linked genes, indicating that it was not added to the X, but was lost from the Y. Its location differs in the maps from the two species, raising the possibility that the X chromosome, as well as the Y, may have been rearranged.

Sex chromosomes have evolved independently in different groups in both the animal and plant kingdoms; however, they share common evolutionary features (Charlesworth 1996). Sex chromosome evolution probably began with the establishment of male-sterility and female- sterility mutations in a region of a pair of autosomes, followed by recombination becoming suppressed in the region, which led to degeneration, differentiation, and extension of male-specific chromosomal regions in evolving XY systems (and female-specific regions in ZW systems). Compared with many animal sex chromosomes, some flowering plant sex chromosomes have evolved recently (Parker 1990), providing opportunities to study the early stages of sex chromosome evolution (for a review, see Jamilena *et al.* 2008). Genetic degeneration is so far not well characterized in plants, and it is difficult to estimate the extent of gene loss from the male-specific region of plant Y chromosomes or to discover the causes of such losses when they occur.

The sex chromosomes of *Spinacia oleracea* and *Asparagus officinalis* evolved very recently, and recombination suppression has either not occurred, or is confined to regions too small to be detected cytologically (Telgmann-Rauber *et al.* 2007; Onodera *et al.* 2011). The sex chromosomes of *Carica papaya* are probably further evolved in Y-chromosome evolution as the YY genotype is inviable, suggesting that genetic degeneration has started (Liu *et al.* 2004) even though the male-specific region of its “Y chromosome” is only 8.4 Mb, or approximately 15% of the chromosome (Ming *et al.* 2008; Na *et al.* 2012). In *Silene latifolia*, the Y chromosome is 50% larger than the X. The strongly heteromorphic sex chromosomes of *S. latifolia* are likely to have evolved long enough ago for some degeneration to have occurred, and, like papaya, the YY genotype is inviable (Westergaard 1958; Janousek *et al.* 1998), so this species may therefore be suitable for studying degeneration. Recent studies of expression levels show that Y-linked genes tend to have lower expression than their X-linked counterparts and also undergo more (presumably deleterious) nonsynonymous substitutions (Bergero and Charlesworth 2011; Chibalina and Filatov 2011), but the ascertainment method used in those studies, based largely on detecting Y-linked variants in cDNA sequences, could not accurately estimate gene losses from the Y, and the “X-only” genes inferred have not yet been shown to have been completely lost from the Y.

The structure of *S. latifolia* sex chromosomes has been characterized using a variety of approaches, including fluorescence *in situ* hybridization to map repetitive sequences on the sex chromosomes and reveal different repetitive sequence compositions of the X and Y chromosomes (for reviews, see Kazama and Matsunaga 2008; Kejnovsky *et al.* 2009). For example, sequences of the X43.1 repetitive family are located at both ends of the X chromosome but only one end of the Y (Buzek *et al.* 1997; Matsunaga *et al.* 1999). Chloroplast sequences have detectably accumulated on the Y chromosome (Kejnovsky *et al.* 2006). Moreover, fluorescence *in situ* hybridization analyses changed the view of the position of pseudoautosomal region of the X chromosome. The pseudoautosomal region of the X chromosome was first characterized as being on the q-arm (Westergaard 1946) but is now known to be located on the p-arm (Lengerova *et al.* 2003; Kazama *et al.* 2006; Kazama and Matsunaga 2008).

Another approach to analyzing the sex chromosomes is isolation of sex-linked genes (Guttman and Charlesworth 1998, Delichere *et al.* 1999; Atanassov *et al.* 2001; Moore *et al.* 2003; Matsunaga *et al.* 2003; Filatov 2004; Bergero *et al.* 2007; Kaiser *et al.* 2009, 2011). Although this can potentially detect genes lost from the Y, most of these gene have both X and Y copies; the one exception for *Slcyt* (Kaiser *et al.* 2009). X-inked genes have been mapped (Filatov 2005; Kaiser *et al.* 2009; Nicolas *et al.* 2005), and their chromosomal arm locations are determined by a combination of laser microdissection and polymerase chain reaction [PCR (Hobza *et al.* 2007)]. Y-linked genes have been physically mapped using Y chromosome deletion mutants, allowing comparisons of the gene order on the X and Y (Lebel-Hardenack *et al.* 2002; Zluvova *et al.* 2007; Ishii *et al.* 2008; Bergero *et al.* 2008). At least three large inversions are proposed to have occurred in the *S. latifolia* Y chromosome during sex chromosome evolution (Kejnovsky and Vyskot 2010).

The isolation of more sex-linked genes will help toward an understanding of the mechanism of sex chromosome degeneration. Floral development differences between *S. latifolia* male and female flowers (Grant *et al.* 1994), and sex-specific expression of some flower development genes (Zluvova *et al.* 2006; Kazama *et al.* 2009), could be due to involvement of genes in the sex determination mechanism, or could be downstream consequences of sex determination. Identifying genes involved in the primary steps in the control of male or female flower development is difficult because these genes must be located in the nonrecombining part of the Y chromosome, so that genetic analysis using mapping is impossible. One way to try and discover candidates for such genes is to clone homologs of such genes from other species and test them for sex-linkage. Indeed, the *SlAP3* gene, a homolog of *APETALA3*, has copies on the *S. latifolia* X and Y chromosomes (Cegan *et al.* 2010; Nishiyama *et al.* 2010)

Here, we identify the *S. latifolia* homolog, *SlWUS1*, of the flower development gene *WUSCHEL* (*WUS*), and demonstrate that *SlWUS1* is sex-linked. In flowering plants, floral organ identity is controlled by the expression and inter-regulation of the ABC genes (Coen and Meyerowitz 1991; Weigel and Meyerowitz 1994) and several other genes (Sablowski 2007). One non-ABC gene, *WUS*, is inferred to be involved in the up-regulation of C-class genes because the size of whorl four is reduced in the *A. thaliana wus* mutant (Laux *et al.* 1996). The *WUS* gene was the first identified gene in the Wuschel-related homeobox (*WOX*) family (Laux *et al.* 1996; Haecker *et al.* 2004). The set of WOX family genes are classified into three separate clades corresponding with different protein functions: the WUS clade, an intermediate clade (sometimes called the WOX clade; the terminology we use in Figure 2), and an ancient clade (van der Graaff *et al.* 2009). In *Arabidopsis thaliana*, proteins of the WUS clade contain a WUS box (TLPLFPMH), and eight genes encoding WOX family proteins have been identified in the genome of this species, including *WUS*. Three *WUS* family members, *WUS*, *WOX5*, and *WOX7*, also encode proteins with an EAR-like (for ERF-associated amphiphilic repression) domain, with the sequence ASLELTLN (van der Graaff *et al.* 2009).

As we detail herein, we have identified the *S. latifolia SlWUS1* gene as an X-linked gene in addition to those already known, and we find that its Y copy has been lost, making it particularly interesting to test whether males express the product at half the level of females, or whether, as in some organisms, dosage compensation has evolved in response the loss of the Y-linked copy. Recently, different two studies tested for dosage compensation in *S. latifolia* using high-throughput sequencing approaches. Chibalina and Filatov (2011) concluded that dosage compensation does not occur, whereas Muyle *et al.* (2012) concluded that it does. Careful studies of individual loci are therefore needed to resolve this and to test whether different genes behave differently (a possibility that must be considered because some mammalian X-linked genes escape inactivation; see Carrel and Willard 2005; Park *et al.* 2010). Moreover, a cytogenetic study found that DNA methylation levels differ between the two X chromosomes in *S. latifolia* females (Vyskot *et al.* 1993), suggesting the possibility that one copy of X-linked genes might be inactivated in this plant, as occurs in the X chromosome inactivation that evolved in mammalian females, probably as a dosage compensation mechanism.

In this article, we identify the *S. latifolia* homolog, *SlWUS1*, of the flower development gene *WUS*, and demonstrate that *SlWUS1* is X-linked but, like *Slcyt*, does not have a Y-linked copy. Together with the mapping data for the *SlWUS1* ortholog to the *Silene vulgaris* linkage group that is homologous with the X chromosome, but probably represents the ancestral state, before the sex chromosomes evolved, the hemizygosity of *SlWUS* in males suggests that this gene does not represent an addition onto the X chromosome. More likely, the *S. latifolia* Y chromosome has undergone a previously undetected rearrangement during sex chromosome evolution, resulting in the loss of this gene. The X is also probably rearranged, although we cannot exclude the possibility that the difference from the X is due to a rearrangement in *S. vulgaris*.

## Materials and Methods

### Plant materials

An inbred *Silene latifolia* line, the K line (Kazama *et al.* 2003), was used in all of our molecular experiments. Plants were grown from seeds in pots in a regulated chamber at 23°. The Berlin (B) plant was obtained from the seed collection of the Royal Botanic Gardens (Kew, United Kingdom). F_1_ progeny for use in segregation analysis were produced by crossing a K line male and a B female plant. For genetic mapping, 90 F_2_ progeny (21 male and 69 female) were generated by crossing a single F_1_ male and female to generate a full-sib family. For sequencing analysis of *S. vulgaris WUS* genes, genomic DNA isolated from the ggK line (Kaiser *et al.* 2011) was used. To genetically map the *SlWUS1* ortholog in *S. vulgaris*, 64 offspring of the SV2 family (Kaiser *et al.* 2009) were used. Leaves and flower buds were frozen in liquid nitrogen and stored at –80° before their use in DNA or RNA preparation.

### Isolation of *S. latifolia WUSCHEL* (*SlWUS*) gene and orthologs in other *Silene* species

Total RNA was extracted from male and female flower buds using an RNeasy Plant Mini Kit (QIAGEN, Hilden, Germany). cDNA was synthesized from 3 mg of total RNA using Superscript II reverse transcriptase (Invitrogen by Life Technologies, Carlsbad, CA). A fraction of the cDNA preparation was then subjected to 30 cycles of PCR amplification (94° for 1 min, 50° for 1 min, and 70° for 1 min) with the degenerate primers 5′-TCY BCR TGC ATN GGR AAT AG-3′ and 5′-TGG TTY CAR AAY CAT AAA GC-3′. The PCR products were then subcloned into the pGEM-T Easy vector (Promega Corporation, Madison, WI) and sequenced using a Big Dye Terminator v. 3.1 Cycle Sequencing Kit (Applied Biosystems by Life Technologies, Foster City, CA) and a 3730xl DNA Analyzer (Applied Biosystems by Life Technologies) with M13 Forward and Reverse primers.

To determine the sequence of the full-length *SlWUS* cDNA, both 5′ and 3′ RACE (Rapid Amplification of cDNA Ends) were performed using GeneRacer (Invitrogen by Life Technologies). Primers for the 5′ and 3′ regions of the isolated fragment were designated as SlWUS1_5RACER1 (5′-CTG CTA ATG CAA GGG AAT ATA GTG TTC TC-3′) and SlWUS1_3RACEF1 (5′-ACA CTT TAC AAT GGA CTT CTA TGG GGA GTA-3′) for *SlWUS1*, and SlWUS2_5RACER1 (5′-GTT CTG AAA GTG AGG ACT TGA GTT TGC AGT-3′) and SlWUS2_3RACEF1 (5′-AGT AGT CAG TGG AAT CCC GTA GGT GGT A-3′) for *SlWUS2*. PCR conditions were 94° for 2 min; five cycles at 94° for 30 sec and 72° for 1 min; five cycles at 94° for 30 sec and 70° for 1 min; 25 cycles at 94° for 30 sec, 65° for 30 sec and 72° for 1 min; and then 72° for 10 min. Nested PCR with SlWUS1_5RACER2 (5′-AAC TTG TAG TAG TGT TAG AGG GAG TAA GC-3′), SlWUS1_3RACEF2 (5′-ACA AGA TCA CTA TGT TGC AGT ATC CGA GA-3′), SlWUS2_5RACER2 (5′-TGC AGT GTT ATT ATT AGT AGG GGG AGT GAG-3′), and SlWUS2_3RACEF2 (5′-CAA GAT CAT CAC TAT GTA GCA CAG CCA GA-3′) primers was carried out using the same PCR conditions. The PCR products were subcloned into the pGEM-T Easy vector and sequenced. Subclones of the 3′ RACE products of *SlWUS1* and *SlWUS2* were used as probes for Southern blot analyses.

To isolate orthologs of *SlWUS* genes, genomic DNA samples were obtained from *S. dioica*, *S. diclinis*, and *S. vulgaris*. For isolation of genomic fragments of *SlWUS1* orthologs, a primer set of SlWUS1F9 and SlWUS1R7 was used in *S. dioica* and *S. diclinis*. SlWUS1F8 and SlWUSR6 were used for isolation of the *SlWUS1* ortholog in *S. vulgaris*. For isolation of genomic fragments of *SlWUS2* orthologs, SlWUS2F9 and SlWUS2R5, SlWUS2F10 and SlWUS2R5, and SlWUS2F10 and SlWUS2 R4 were used in *S. dioica*, *S. diclinis*, and *S. vulgaris*, respectively. The products were cloned as described above, then sequenced using sequencing primers. All primes used for the sequencing analysis are listed in supporting information, Table S1.

### Phylogenetic tree analysis

The amino acid sequences of the WUSCHEL homeobox proteins were aligned using CLUSTAL X version 1.81 (Thompson *et al.* 1997). The alignment was used in a neighbor-joining (NJ) analysis (Saitou and Nei 1987) in the software MEGA4 (Tamura *et al.* 2007). The resulting tree was tested by 1000 bootstrap replications using the MEGA program. For the phylogenetic analysis of the *SlWUS1* and *SlWUS2* orthologs in *Silene* species (Figure 6), the deduced amino acid sequences were aligned using the corresponding amino acid sequences of SlWUS1 and SlWUS2 as guides. The alignment was also used in NJ analyses using MEGA4 software.

### Southern blot analysis

Genomic DNA was extracted from *S. latifolia* leaves using the Nucleon PhytoPure Genomic DNA Extraction Kit (GE Healthcare UK Ltd, Buckinghamshire, England). Genomic DNA (15 µg) was digested with *Eco*RV or *Hin*dIII for 12 h. The concentrations of the digests were measured and loaded equally onto a 1.0% (w/v) agarose gel and then transferred to an Immobilon-Ny+ membrane (Millipore). For the analysis shown in Figure 3A, inserts of subclones of 3′ RACE products of *SlWUS1* and *SlWUS2* were used as probes. For the analysis shown in Figure S1, the conserved homeo domain regions were amplified using *SlWUS1*-specific primer set (5′-AGT AGC ACA AGG TGG ACA CCT ACA A-3′ and 5′-CTT GTA GTA GTG TTA GAG GGA GTA-3′) and *SlWUS2*-specific primer set (5′-AGT AGC ACA AGG TGG ACA CCG ACG A-3′ and 5′-AGT GAG GAC TTG AGT TTG CAG TG-3′) and used as probes. Hybridization and detection were carried out as described previously (Kazama *et al.* 2009). Hybridization and signal detection were performed using the Gene Images AlkPhos Direct Labeling and Detection System (GE Healthcare UK Ltd). Posthybridization washes were carried out under relatively high stringency conditions at 60° for 2 × 10 min (Figure 3A) and low stringency conditions at 50° for 2 × 10 min (Figure S1). The hybridized membranes were visualized using Hyperfilm ECL (GE Healthcare UK Ltd) at room temperature with appropriate exposure times.

### Expression analysis

RNA purification and cDNA synthesis were performed as described previously. Quantitative RT-PCR (qRT-PCR) was performed using a LightCycler instrument (Roche Diagnostics GmbH, Mannheim, Germany) with SYBR-Green detection chemistry (Applied Biosystems by Life Technologies). The *SlWUS1*-specific primers (5′-TTG TTCT CGT CTT CGC CGC TA-3′and 5′-AGG AAG TGT CTC TAT CTC GG-3′) and the *SlWUS2*-specific primers (5′-CAA CTC CTA CGG ATA TGG CTG-3′and 5′-CCA TCA TCG GGT CTT GTT CC-3′) were used to amplify a fragment. 18S rRNA was used as a reference control by performing QRT-PCR with 100,000-fold cDNA dilutions as templates in each sample. The 18S rRNA-specific primes were as follows: 5′-GGC AAC GGA TAT CTC GGC TCT C-3′ and 5′-TGA CGC CCA GGC AGA CGT GC-3′. Primer specificity was confirmed by sequencing each fragment after qRT-PCR. The qRT-PCR data shown are average relative quantities ± SE from at least three biological replicates. RT-PCR and qRT-PCR for allele-specific expression analysis were performed by the same method using K-line specific primers (5′-AAG AAA AGG CTT ACT CCC TCT AAC A-3′ and 5′-AGT ACT CCC CAT AGA AGT CCA TTG T-3′) and B-line−specific primers (5′-AAG AAA AGG CTT ACT CCC TCA AAC T-3′ and 5′-AGT ACT CCC CAT AGA AGT CCA CTG G T-3′). In Figure 4B, primers specific for the *S. latifolia* actin gene (5′-TTA CCG TAA AGG TCC TTC CTG AT-3′ and 5′-AGC TTC GTG TTG CTC CTG AAG A-3′) were used as a control.

### Testing for sex linkage and genetic mapping

Genomic DNA fragments of *SlWUS1* and five other previously known X-linked genes (*SlX1*, *SlX3*, *SlX4*, *SlX7*, and *DD44X*) were amplified by PCR using the primers listed in Table S2. PCR products were cloned using the TOPO TA Cloning Kit (Invitrogen by Life Technologies) and sequenced. Using polymorphisms differing between the K line and B parent sequences, we designed primers that amplified only the K-line sequences for *SlWUS1* (5′-GAG AAC AAG TTT AGG GTA AGT ATG-3′ and 5′-AGT TAT CTC TAA ATG ATA CTC CGT A-3′) and *SlWUS2* (5′-AGT ATG TCT CAA ACA AGC AGT CAG-3′ and 5′-ACT CCT CCT GAT ATT GAA CAG TC-3′). PCR with the primers specific for the K line parent was then performed on F_1_ male and female progeny, allowing us to score the segregation of these genes. The PCR parameters used were 94° for 3 min, followed by 33 cycles at 94° for 30 sec, 53° for 30 s, and 72° for 30 sec.

To construct the X map, primers for high-resolution melting curve (HRM) analysis were designed for *SlWUS1*, *SlX1*, *SlX3*, *SlX4*, *SlX7*, and *DD44X*. The primers were then tested by HRM using genomic DNAs of the K-line parent, the B parent, and F_1_ plants. HRM was performed using a LightCycler 480s (Roche Diagnostics GmbH) as described previously (Kazama *et al.* 2011). Among the primer sets designed, the primer sets for *SlWUS1*, *SlX1*, *SlX3*, *SlX4*, and *DD44X* could distinguish polymorphisms differing between the K-line parent and the B parent. These primers were used for genotyping analysis (Table S2). To genotype *SlX3*, PCR primers for a simple sequence-length polymorphism marker were designed (5′-CAA TGA AGC TTC GTC CAC TGT TGA-3′ and 5′-ATC CTA CGG CGG TTT AGT TCG GA-3′). The PCR parameters were 94° for 5 min, followed by 30 cycles at 94° for 30 sec, 60° for 30 sec, and 72° for 60 sec. Amplified fragments were separated by agarose gel electrophoresis (1%). The K-line derived fragment was 749 bp, whereas fragment inherited from the B parent was 940 bp. To genotype *SlX7*, a cleaved amplified polymorphic sequence marker was designed. Genomic DNA fragments were amplified by PCR using gene-specific primers for *SlX7* (5′-TTC CTT GAT GCA CCA AGT GAT T-3′ and 5′-CCA GCA AAT ACA ACT CCC TTC T-3′). The PCR parameters were 94° for 5 min, followed by 30 cycles at 94° for 30 sec, 60° for 30 sec, and 72° for 30 sec. The amplified fragments were digested by *Hinc*II and separated by agarose gel electrophoresis (1%). Another cleaved amplified polymorphic sequence marker was also designed to map the *SlWUS1* ortholog in *S. vulgaris* (*SvWUS1*). A fragment was amplified Phusion enzyme (Finnzymes, Espoo, Finland) in a PIKO 24 thermal cycler (Finnzymes) using primers specific for *SvWUS1* (5′-TTT GTT CTC GTC TTC GCC GCT ATG-3′ and 5′-GTG TTG TTC ACA TTT CCA GTA CC-3′). The PCR conditions were as follows: 40 sec at 98°, followed by 10 cycles of 5 sec at 98, 5 s at 63°, 1 min at 72°; 25 cycles of 5 sec at 98°, 5 sec at 63°, 100 s at 72°; final extension at 72° for 5 min). The amplified fragments were digested by *Rsa*I and separated by agarose gel electrophoresis (1.5%). The Kosambi mapping function was used for computing genetic distances in centimorgans (Kosambi 1944).

## Results

### Identification of *WUS* homologs in *S. latifolia*

**Figure 1:**
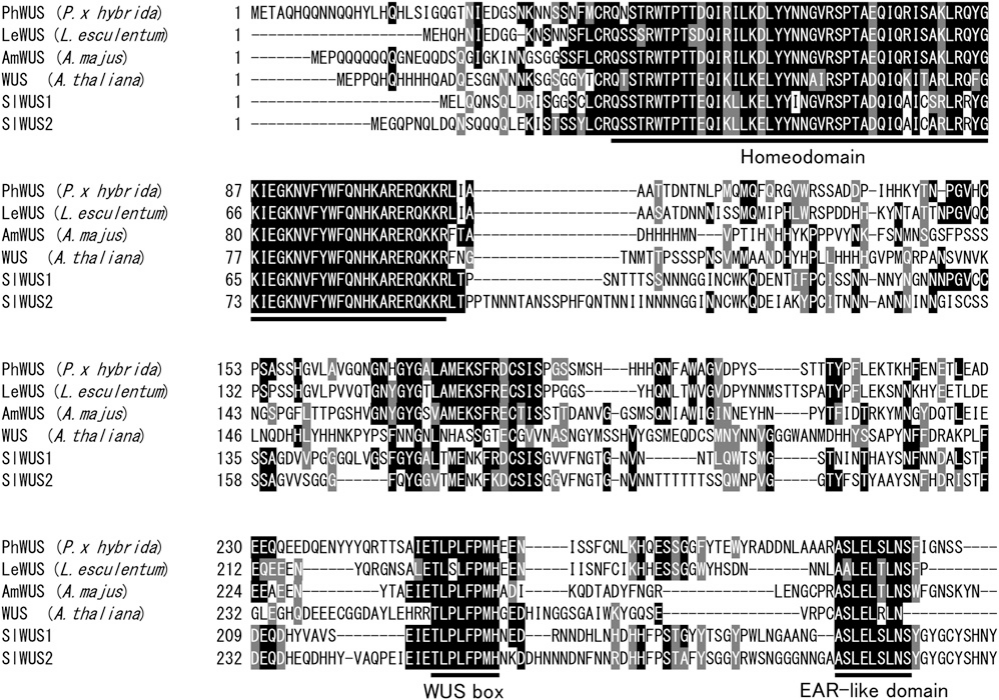
ClustalW alignment of amino acid sequences of SlWUS and other plant WUS proteins. Identical residues are highlighted in black and similar residues in gray. Dashes represent gaps in the alignment. The conserved homeodomain region, the WUS box, and the EAR-like domain are indicated.

Using degenerate primers based on the *A. thaliana WUS* sequence, we performed PCR on *S. latifolia* male and female cDNAs, which amplified two fragments: 475 bp and 539 bp. A BLAST search identified homologous sequences with the *A. thaliana WUS* sequence (e-values: 0.01 and 0.013). The full-length cDNA sequences of the amplified fragments (using RACE) were a 1212-bp cDNA encoding a protein of 282 amino acid residues and a 1283 bp encoding a 317-amino acid protein. The former cDNA (which we named *SlWUS1*) has 67.5% amino acid identity with the *A. thaliana WUS* sequence and the latter (*SlWUS2*) 55.8%. Homologs of the gene classified into the WUS-clade have been identified in many flowering plants, and the sequences are similar, not only in the homeobox domain but also in other regions. Alignment of the deduced amino acid sequences with WUS homologs in other plants showed high conservation of the homeobox domain, and also the C-terminal domains (Figure 1), which include an acidic domain (Mayer *et al.* 1998), the WUS box (TLPLFPMH; Haecker *et al.* 2004), and the EAR-like domain (ASLELTLN; Ohta *et al.* 2001).

**Figure 2:**
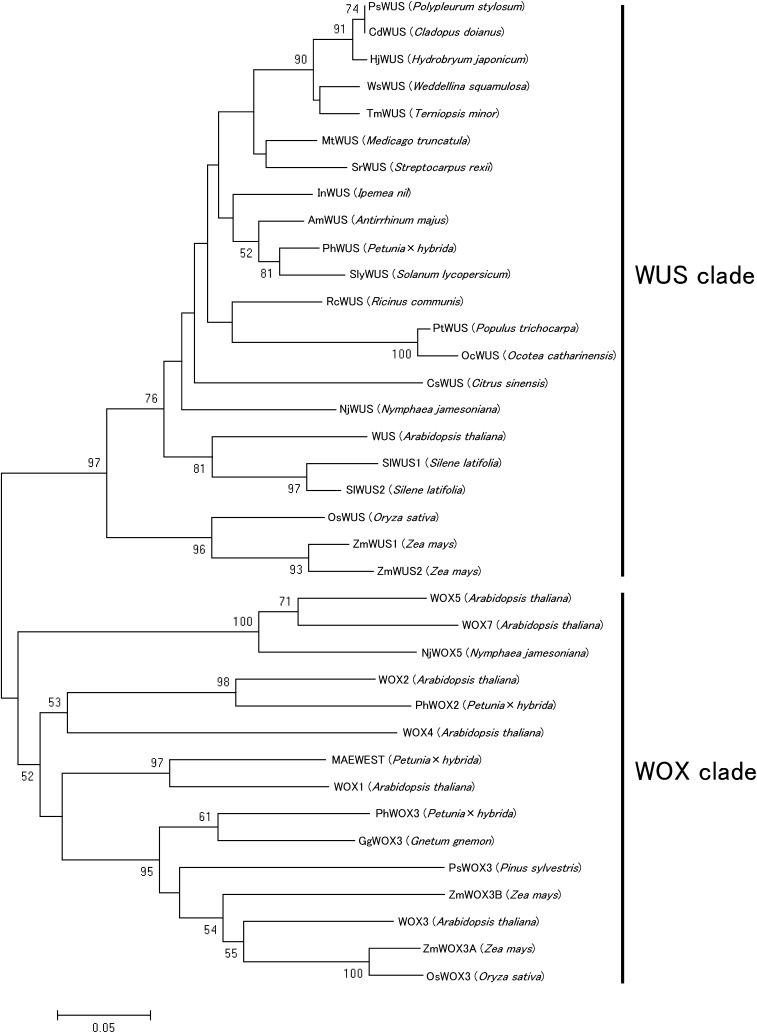
Phylogenetic tree of WUSCHEL-related homeobox genes [the ancient clade of (van der Graaff *et al.* 2009) is not shown]. The tree was constructed by the NJ method (Saitou and Nei 1987). Branch lengths are proportional to evolutionary distances. Bootstrap values, based on 1000 replications, are shown only when >50%. The GenBank accession numbers of the amino acid sequences are as follows: PhWUS (Q8LL11.1), InWUS (ACD62900.1), RcWUS (XP_002530735), MtWUS (ACK77479.1), AmWUS (Q6YBV1.1), SlyWUS (ADZ13564), SrWUS (ABS01330.1), TmWUS (BAJ10718.1), CsWUS (ABS84661.1), NjWUS (CAT03215.1), PsWUS (BAJ10712.1), WsWUS (BAJ10714.1), PtWUS (CAJ84139.1), HjWUS (BAJ10706.1), CdWUS (NP_565429.1), WUS (CAA09986.1), OcWUS (ACN56438.1), OsWUS (BAE48303.1), ZmWUS1 (NP_001105960.1), ZmWUS2 (NP_001105961.1), MAEWEST (ACA64093.1), WOX1 (NP_188428), WOX2 (NP_200742.2), GgWOX3 (CAT02931.1), PsWOX3 (CAT02936.1), PhWOX2 (ACA64094.1), WOX5 (NP_187735.2), NjWOX5 (CAT03216.1), WOX3 (NP_180429), PhWOX3 (ACU68503.1), ZmWOX3B (NP_001106240), ZmWOX3A (NP_001105160.1), OsWOX3 (Q33DK1.1), WOX4 (NP_175145.2), and WOX7 (NP_196196.1). Both SlWUS sequences fell into the clade with other plants’ WUS homologs, not the WOX clade.

Our NJ tree showed that both *SlWUS* sequences belong to the clade that included the WUS homologs of other plants, not to the WOX gene clade (see *Introduction* and Figure 2). This result suggests that *SlWUS1* and *SlWUS2* are orthologs of the *A. thaliana WUSCHEL* gene. The synonymous divergence between the two *S. latifolia* sequences is 0.628 (using the method of Pamilo and Bianchi (1993); nonsynonymous divergence is 0.149). *SlWUS1* and *SlWUS2* therefore diverged long before the sex chromosomes started diverging; silent-site divergence estimates between the sequences of many *S. latifolia* X- and Y-linked gene pairs range from 1.7% (for *SlX1*/*SlY1*, at one end of our X genetic map) to 16% [for *SlX4*/*SlY4*, which is distal in the X map; see Filatov and Charlesworth (2002) and Nicolas *et al.* (2005)], and the largest synonymous divergence values for gene pairs with X- and Y-linked copies is around 25%. The divergence time of the duplicates also greatly predates that between *S. latifolia* and *S. vulgaris*, a species used below as an outgroup; based on 12 gene sequences, silent divergence values between orthologs of these two species never exceeds 20%, and the mean is 11.2% (standard error 1.3%).

### Sex-linkage of the *SlWUS1* gene

**Figure 3:**
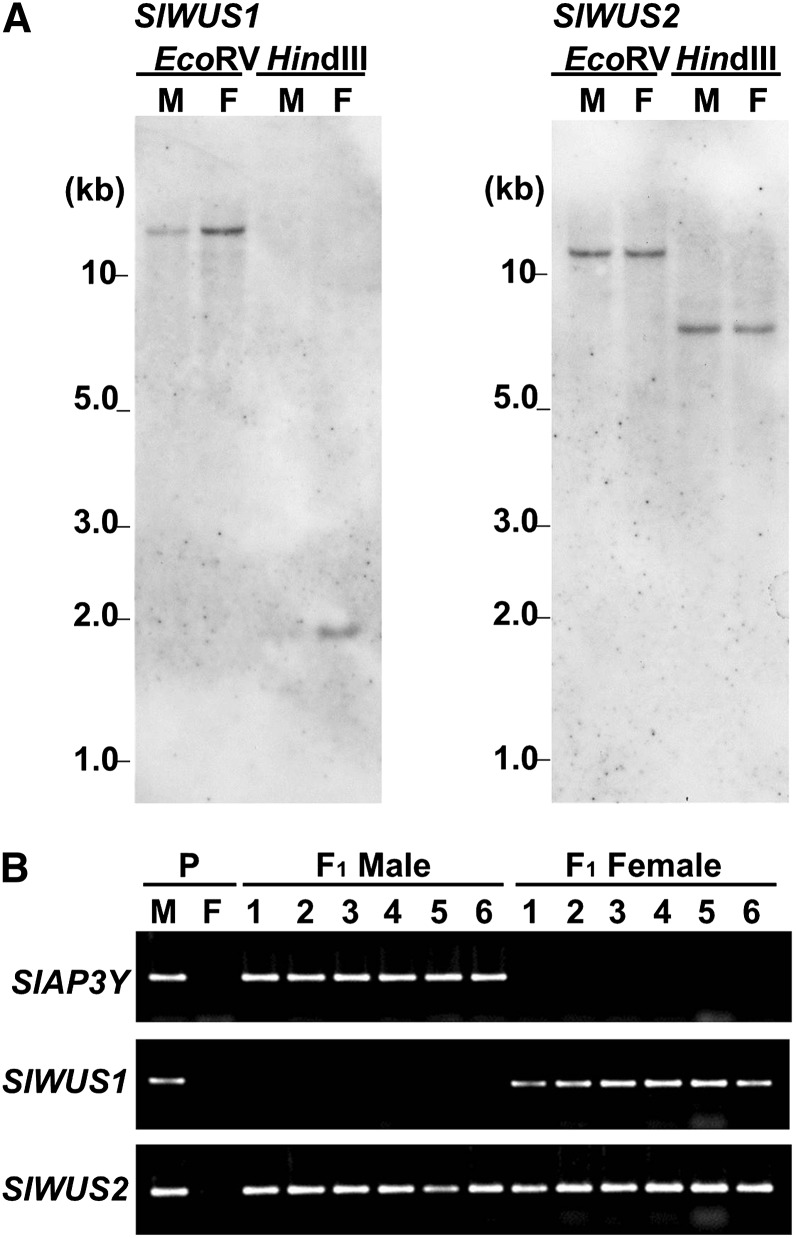
X-linkage analysis of *SlWUS1*. (A) Southern blot analysis of *SlWUS* genes. Genomic DNA was isolated from leaves of *S. latifolia* male and female plants, and digested with *Eco*RV and *Hin*dIII. Each lane shows a single signal. There is no difference in band sizes between males and females for either gene and no difference in signal intensity for *SlWUS2*, but the signal in females was stronger than that in males for *SlWUS1*. M, male; F, female. (B) Segregation analysis of the *SlWUS* genes using PCR with allele-specific primers. DNA was isolated from interstrain-crossing parents (K-line male and B-line female) and their progeny. Primers were designed from the K-line− specific sequence. The upper panel shows segregation of *SlAP3Y* as the Y-linked control (Matsunaga *et al.* 2003). The middle panel and lower panels show the segregation of *SlWUS1* and *SlWUS2*, respectively. P, parents of interstrain crossing (K-line male and B-line female). Numbers indicate the numbers of individuals tested (1–6).

To determine whether *SlWUS1* or *SlWUS2* is located on the sex chromosomes, we used genomic Southern hybridization analyses of these genes. Specific regions from each gene, downstream of the conserved homeodomain sequences, were used as probes. The *SlWUS1*-specific probe gave weaker signals with males than females (Figure 3A), suggesting that *SlWUS1* may be located on the X chromosome. In contrast, the *SlWUS2*-specific probe showed equal signal intensities in both sexes. To further test X-linkage of *SlWUS1*, segregation analysis was performed on F_1_ progeny from the cross between a K line male and a B female parent (see *Materials and Methods*). PCR on F_1_ male and female plants was performed using primers specific for the K line sequences. The F_1_ males X chromosome is inherited from the B female parent and their Y chromosome from the K line male, whereas the F_1_ females have one X chromosome from the B parent and the other from the K line. *SlWUS1* amplified only from females, whereas *SlWUS2* amplified from progeny of both sexes (Figure 3B). The *SlAP3Y* gene, a known Y-linked gene (Matsunaga *et al.* 2003), also amplified only from the F_1_ males. We conclude that *SlWUS1* is X-linked and *SlWUS2* is autosomal.

To test whether there is also a Y-linked copy of *SlWUS1*, we did low-stringency Southern blot analyses using probes complementary to the highly conserved homeodomain regions. Both *SlWUS1* and *SlWUS2* were detected, and the *SlWUS1* probe and the *SlWUS2* probe cross-hybridize weakly with one another; the nucleotide divergence between these two homeodomain region probes (which we estimated using all site types, not just synonymous ones, because the sequences are too short for accurate synonymous site estimates) is 16%. The divergence between the *SlWUS1* sequence and its Y-linked copy, if one exists, is probably similar to this value, based on the genetic map location of *SlWUS1* close to the *SlX3* gene (see the section *Genetic mapping of the X-linked SlWUS1 gene on the X chromosome and the ancestral chromosome*), in the older evolutionary stratum of Bergero *et al.* (2007), with silent- or synonymous-site divergence up to 25% (see the section *Identification of WUS homologs in S. latifolia*). Because total divergence will be less than silent-site divergence, this experiment should thus be able to detect a Y copy, if it exists. However, no male-specific signal was detected (Figure S1). We conclude that there is no homologous *SlWUS1* copy on the Y chromosome. Below, we discuss the divergence.

To further test whether a homologous Y-linked *SlWUS1* copy exists, we used genomic DNA and cDNA from male individuals of *S. latifolia* and two other closely related dioecious species, *S. dioica* and *S. diclinis*, in PCR amplification tests using primers designed to match regions of the homeodomain that are conserved between *SlWUS1* and *SlWUS2* in *S. latifolia* and the *A. thaliana* sequence. All amplicons were sequenced and only the two *SlWUS1* and *SlWUS2* copies were detected (Figure S2). We conclude that *SlWUS1* has no homologous Y-linked copy.

### Expression of *SlWUS1* in flower buds and leaves and tests for sex differences in expression and dosage compensation

**Figure 4:**
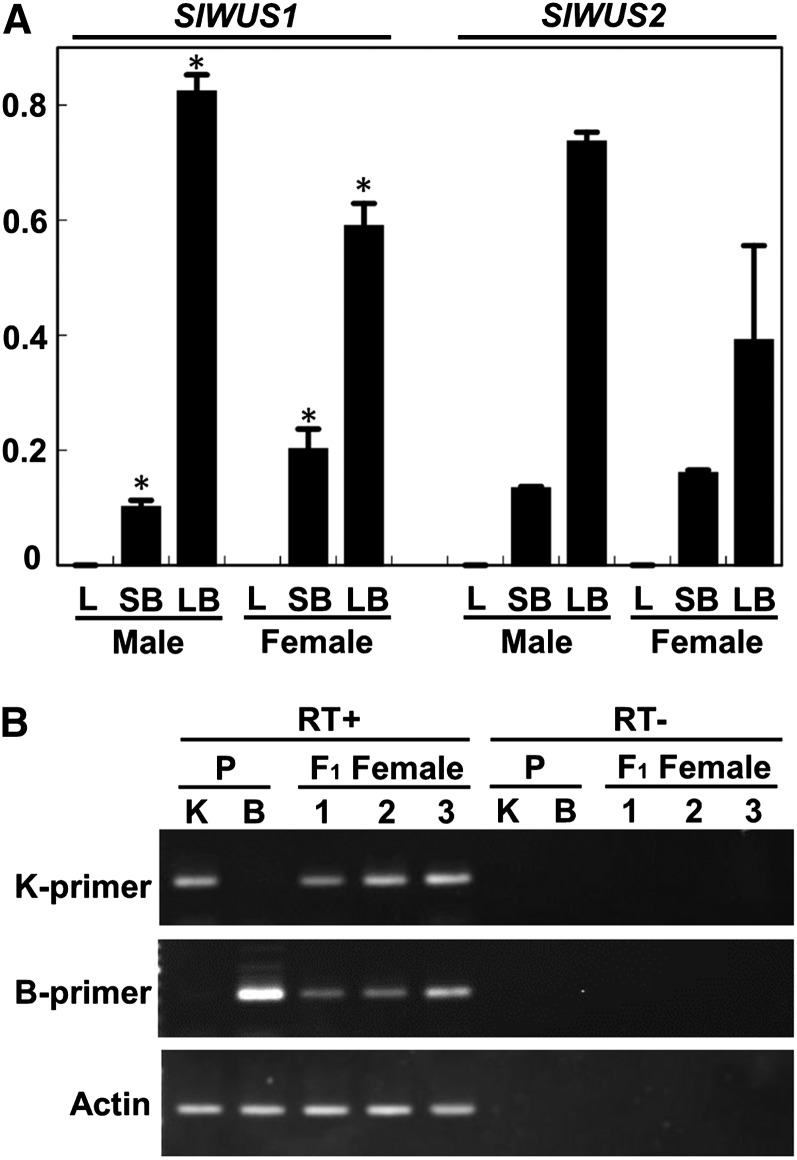
Expression analyses of *SlWUS*. (A) qRT-PCR analysis of the *SlWUS* genes. Total RNA was isolated from leaves and buds at stages 1–8 (<1 mm, small flower buds) and from buds at stages 9–11 (2–5 mm, large flower buds) as defined by Grant *et al.* (1994) and analyzed by QRT-PCR using *SlWUS1*- and *SlWUS2*-specific primers, respectively. 18S rRNA was used as a reference control by perfoming QRT-PCR with 100,000-fold cDNA dilutions as templates in each sample. Both *SlWUS1* and *SlWUS2* were expressed in flower buds. Significant differences of *SlWUS1* expressions between males and females in each stage based on Student’s *t*-test at *P* < 0.05 were indicated by asterisks. L, leaves, SB, small flower buds at stages 1−8, LB, large flower buds at stage 9−11. (B) RT-PCR analysis of the *SlWUS1* gene using ecotype-specific primers. RNAs were isolated from female flower buds (<1 mm) of the K-line, B-line, and F_1_ individuals. RT-PCR was performed using K-line and B-line specific primers, respectively. In the K-line and B-line, each primer set amplified *SlWUS1* fragments in an ecotype-specific manner. In the F_1_ females, both K-line− and B-line−derived copies were amplified. An actin gene was amplified as a control.

To examine the tissues expressing *SlWUS1* and *SlWUS2*, qRT-PCR analyses were performed with *SlWUS1*- and *SlWUS2*-specific primers (Figure 4A) using RNA extracted from leaves and flower buds of male and female plants corresponding to stages 1−8 (small flower buds) and 9−11 (large flower buds). The *SlWUS* transcript levels, relative to the 18S rRNA control, were calculated from at least three independent experiments. We used this small tissue panel because we focused on *WUS* function(s) that might be involved in floral development in *S. latifolia*. Both *SlWUS1* and *SlWUS2* were expressed in developing flower buds of both sexes but not in leaves. Both genes showed greater expression levels in large flower buds than the small ones. In the small flower bud sample, females showed greater *SlWUS1* expression than males (*P* < 0.05, Student’s *t*-test). In contrast, large female buds tended to show lower expression levels of *SlWUS1* and *SlWUS2* than large male ones, although the difference for *SlWUS2* transcription level was not statistically significant. The somewhat-lower expression levels of both *SlWUS* genes observed in large female flower buds, compared with male ones, is consistent with the previous report that *Arabidopsis WUSCHEL* is required for anther development (Deyhle *et al.* 2007).

Due to the complete X-linkage of *SlWUS1*, with no Y-linked copy, the *S. latifolia* male genome has one *SlWUS1*copy, whereas females have two. We therefore tested whether dosage compensation occurs to increase expression in males. First, we found roughly 2-fold greater expression level of *SlWUS1* in small female flower buds than male ones and therefore concluded that the X-linked *SlWUS1* gene is not expressed from only one of the females’ alleles, whereas the other is inactivated. We further examined expression of each X-linked *SlWUS1* allele in F_1_ females from an interstrain cross between the K-line male and a B female parent by RT-PCR, using K-line and B parent-specific primers. Both parental *SlWUS1* X-linked alleles were detected in the female progeny (Figure 4B). Finally, we also checked the expression levels of both alleles by qRT-PCR. The expression levels did not differ significantly (Table S3).

These results indicate that there is no plant-wide inactivation of one *SlWUS1* allele in *S. latifolia* females, as has been suggested from the observed methylation of one of the two X chromosomes (see *Introduction*). These results do not, of course, exclude random X-inactivation in individual cells, or sets of cells within the buds studied (like the situation in mammalian females). With such inactivation, both alleles would still be expressed. However, it seems incompatible with the much greater expression of *SlWUS1* in small female flower buds than male ones; there is therefore no evidence that this gene is dosage compensated, although it cannot be ruled out that dosage compensation might have evolved, increasing expression in males, but that expression in female buds is also greatly increased, perhaps because the gene has a function specific to female buds.

### Genetic mapping of the X-linked *SlWUS1* gene on the X chromosome and the ancestral chromosome

**Figure 5:**
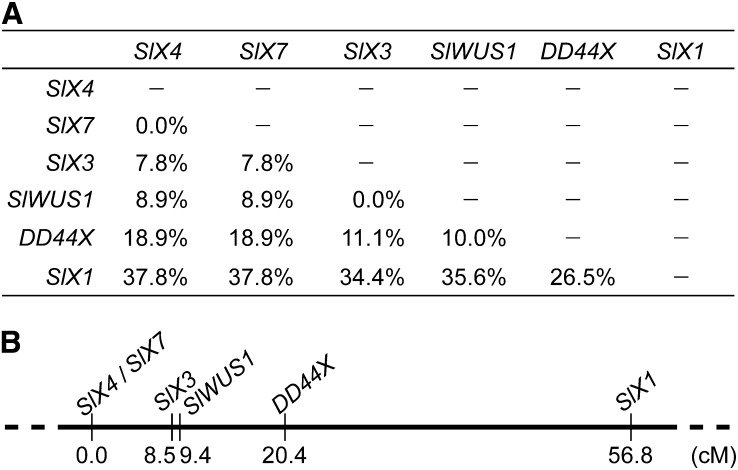
Genetic map of six X-linked genes in *S. latifolia*. Recombination rates between the genes in this study are shown (A). (B) Genetic map; genetic distances were estimated using the Kosambi mapping function (Kosambi 1944). *SlWUS1* is closely linked to *SlX3*.

We genetically mapped *SlWUS1* using segregation analysis of 90 F_2_ progeny from the interstrain cross between a K-line male and a B female parent. We identified haplotypes of *SlWUS1* and several other known X-linked genes, *SlX1* (Delichere *et al.* 1999), *SlX3* (Nicolas *et al.* 2005), *SlX4* (Atanassov *et al.* 2001), *DD44X* (Moore *et al.* 2003), and *SlX7* (Bergero *et al.* 2007) in the K-line and the B parent, and used them in segregation analysis. Recombination rates and a genetic map of the X chromosome are shown in Figure 5. *SlWUS1* maps in the middle of the X chromosome, 0.9 cM from *SlX3* and 11.0 cM from *DD44X* (in order of distance from *SlX4*, the gene order is *SlX7*, *SlX3*, *SlWUS1*, *DD44X*, and *SlX1*; see Figure 5) with only one recombinant found between *SlWUS1* and *SlX3* among the offspring scored. The breakpoints of two Y chromosome inversions have been identified as being in regions near the *SlX3*/*SlY3* genes, by detecting markers lost in plants with Y chromosome deletions (Zluvova *et al.* 2007; Bergero *et al.* 2008). This suggests that these regions, including the lost Y copy of *SlWUS1*, and perhaps other genes, could have been deleted during the inversions.

An alternative possibility to explain why there is no Y-linked copy of *SlWUS1* is that it *SlWUS1* was translocated onto the X chromosome from an autosome after the sex chromosomes evolved. To test between these alternatives, we genetically mapped the *SlWUS1* ortholog in the related species *S. vulgaris* (*SvWUS1*), which has no sex chromosomes, using 64 F_2_ progeny of the mapping family SV2 (Kaiser *et al.* 2009). The map will be presented in a forthcoming publication. *SvWUS1* mapped on the chromosome that carries the orthologs of the other fully X-linked genes, so we conclude that the Y copy of *SlWUS1* was deleted after the sex chromosomes evolved. In *S. vulgaris* this gene does map not close to the ortholog of *SlX3*, but 8.5 cM from *SvCyp*, and 24.7 cM from *SvXY3*, suggesting that the *S. latifolia* X chromosome may be rearranged.

**Figure 6:**
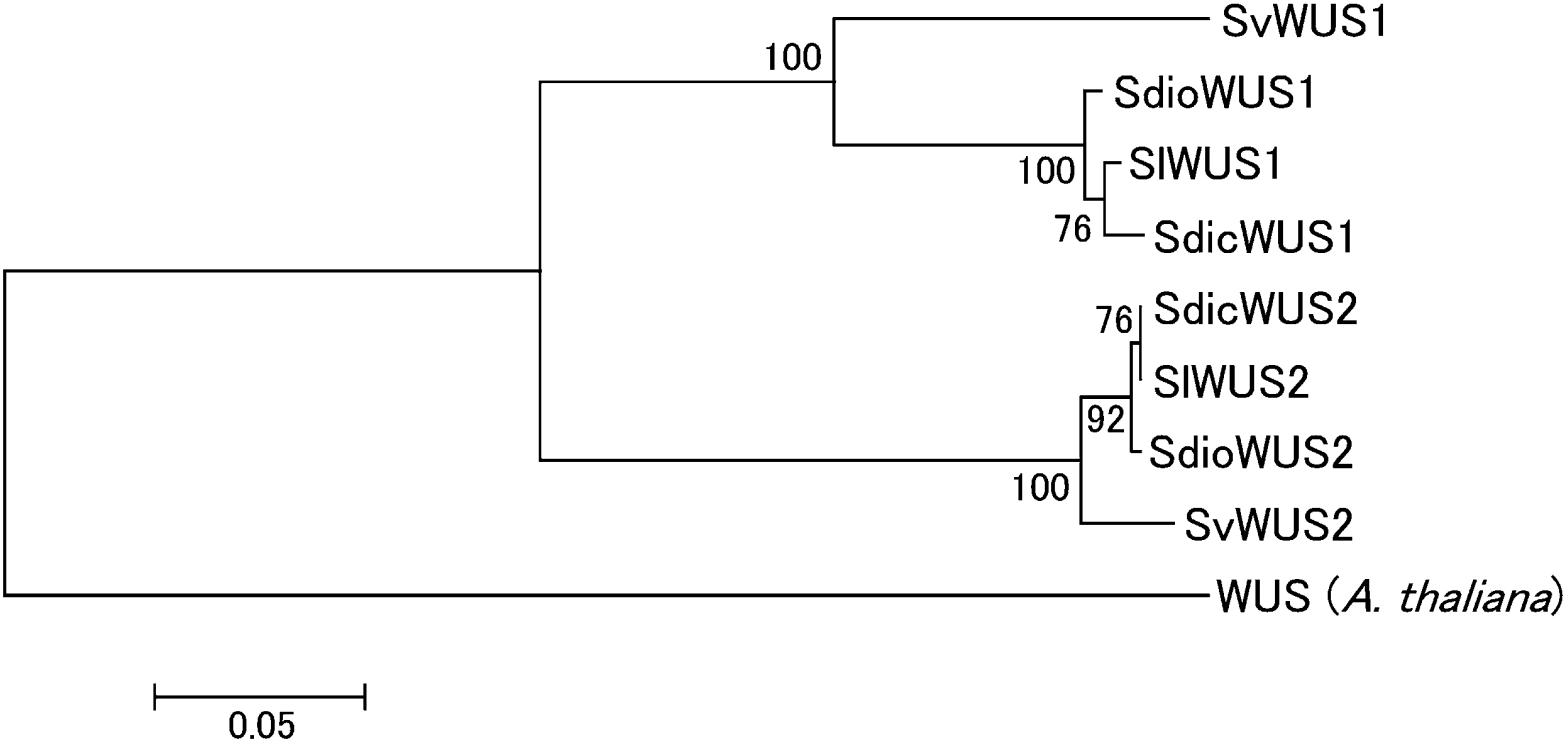
NJ tree (Saitou and Nei 1987) of WUS homologs in *Silene* species. The tree was estimated using an amino acid alignment of cDNA sequence from *S. latifolia* and a genomic sequence from *S. vulgaris*, or using both (in *S. dioica* and *S. diclinis*). The *A. thaliana* WUS amino acids were used as an out-group. Bootstrap values, based on 1000 replications, are shown.

We sequenced the *S. vulgaris* ortholog (*SvWUS2*) and constructed a phylogenetic tree using the *SlWUS1* and *SlWUS2* ortholog sequences in the *Silene* species studied (Figure 6). This confirms our conclusion from the divergence data (see the section *Identification of WUS homologs in S. latifolia*) that *SlWUS1* and *SlWUS2* duplicated long before the split between the ancestors of *S. latifolia* and *S. vulgaris*. We therefore did not map *SlWUS2*, since its location is not relevant for the questions we study here.

## Discussion

Despite their sequence divergence, degenerate primers can sometimes be used to identify genes in *S. latifolia* using *A. thaliana* sequences. We used this approach to sequence and study *SlWUS1*, a new X-linked gene in *S. latifolia*. Although the *A. thaliana* homolog is important in flower development, it may not be related to sex determination in *S. latifolia*. The *SlWUS1* is X-linked, but has no homologous Y-linked copy, and sex is genetically determined in *S. latifolia* by a strongly male-determining Y chromosome (Westergaard 1958). This is only the third sex-linked gene with no Y homolog known from this plant. There is currently a strong bias toward discovering plant sex-linked genes through transcripts from Y-linked alleles, leading to underestimation of the extent of gene loss. However, loss of genes from plant Y chromosomes may really be much less than in animals, because some Y-linked genes will be under selection pressure to preserve their function because they are expressed and have important functions in haploid male gametophytes. Of the three genes so far known without Y copies, the function of the *SlCyt* gene is not currently known. Although the Y copy of the *MROS3* gene is nonfunctional, this gene is expressed only in developing male flowers, so there is no reason to expect selection on *MROS3-Y* in pollen (Guttman and Charlesworth 1998).

Each example, of a plant X-linked gene without a Y copy is of great interest because these genes are the most relevant for testing whether dosage compensation has evolved in these young sex chromosomes. There is no X-inactivation of *SlWUS1* in females, despite the observed apparent methylation of one of the X chromosomes, and no evidence for dosage compensation (small male buds have considerably lower expression than female ones, see Results). To the extent that a single gene is informative, this finding is consistent with the conclusion that dosage compensation has not yet evolved in the *S. latifolia* sex chromosomes (Chibalina and Filatov 2011).

*SlWUS1* appears to have been lost from the Y chromosome during the evolution of dioecy. However, it is not known whether this was a degenerative loss, *i.e.*, a deleterious change that would select for dosage compensation. Assuming that *SlWUS1* has a function similar to the *Arabidopsis WUSCHEL* (See results and Figure 1 and Figure 2), loss of the gene from the Y chromosome creates a dosage difference between the sexes, which might lead to differences in floral phenotypes between male and female flowers. In *A. thaliana*, *WUSCHEL* is expressed in developing anthers and is required for development of the stomium (Deyhle *et al.* 2007). Although expression in anthers might suggest that loss of the *SlWUS1Y* copy is likely to be deleterious (predicting that dosage compensation should evolve), it also complicates investigation of sex differences in the dosage of *SlWUS1* in large flower buds because only male flower buds have developing anthers at these stages.

It remains possible, however, that the loss of the *SlWUS1Y* copy was adaptive. In *S. latifolia*, a sex difference in the size of floral whorl four is seen early in development, with a smaller size in males than females (Grant *et al.* 1994), consistent with the reduced size of whorl four in the *A. thaliana wus* mutant (Laux *et al.* 1996). Our qRT-PCR (Figure 4A) showed that female flower buds express *SlWUS1* at twice the level in male buds in stages 1−8, and the dosage difference may lead to the reduced gynoecium size of male flowers. This may be beneficial in *S. latifolia* males, releasing resources that allow them to produce more flowers, thus becoming more attractive to pollinators and increasing male reproduction success. Under such an adaptive hypothesis for the loss of the *SlWUS1Y* copy, dosage compensation would not be predicted to evolve for the *SlWUS1* gene.

Alternatively, the *SlWUS1* copy may have evolved a function in female flower development and have evolved elevated expression in female buds that more than compensates for any increase in males (assuming that dosage compensation has evolved, increasing male expression). A *S. latifolia* homolog of the *CLAVATA1* gene is differentially expressed in male and female flower development (Koizumi *et al.* 2010). *CLAVATA1* is involved in stem cell maintenance in shoot and flower meristems together with *WUS* in *A. thaliana* (Brand *et al.* 2000; Ogawa *et al.* 2008). It is possible that *SlWUS1* contributes to female flower development through the *WUSCHEL-CLAVATA* pathway.

The mechanism by which the Y copy of *SlWUS1* has disappeared could be deletion of an entire Y region, deleting several or many genes, or by a specific deletion of just this gene, or by loss-of-function mutations followed by sequence divergence, so that no Y-linked copy remains detectable even in our low-stringency Southern blotting experiments, including in *S. dioica* and *S. diclinis* (see *Results*). We therefore conclude that the Y-linked copy of the *SlWUS1* gene was lost before these species diverged, in a deletion event in their common ancestor, before these dioecious species split, additional to the inversions currently detected (Kejnovsky and Vyskot 2010).

Because our results exclude the possibility that *SlWUS1* was translocated to the X chromosome from an autosome after the sex chromosomes evolved, they suggest that gene losses are occurring, an important form of genetic degeneration that has not previously been documented in the *S. latifolia* Y. An addition to the X was reported for *SlCyt*, based on evidence that the *S. vulgaris* ortholog is unlinked to the other sex chromosome homologs in that species (Kaiser *et al.* 2009). However, genetic mapping has subsequently shown that this was due to a gap in the genetic map, and that all genes that are fully sex-linked in *S. latifolia* map to a single *S. vulgaris* linkage group (R. Bergero, S. Qiu, A. Forrest, H. Borthwick, and D. Charlesworth, unpublished results).

Farther mapping of sex-linked genes will be needed to test whether the loss of the Y copy involved deletion of other genes and to learn the details of the rearrangement during sex chromosome evolution in *S. latifolia* and its close relatives. Deleted genes would have to have been dispensable, either because they are expressed in the sporophyte stage (as just discussed), or else because the chromosome must have carried genes that were already inactive, so that their function was adequately carried out by a single X-linked copy. In *A. thaliana*, the *WUSCHEL* gene is required for both meristem size regulation of whorl 4, and for anther development (Deyhle *et al.* 2007), making it unlikely that this male function is dispensable in *S. latifolia*.

## Supplementary Material

Supporting Information
