# Supplementary material for: SlWUS1; An X-linked Gene Having No Homologous Y-Linked Copy in Silene latifolia
Source: G3 (Bethesda). 2012 Oct 1;2(10):1269–78. doi: 10.1534/g3.112.003749 (PMC3464119; doi:10.1534/g3.112.003749)
Supplement: Supporting Information [file supp_2_10_1269__index.html]

Supporting Information 

# *SlWUS1*; An X-linked Gene Having No Homologous Y-Linked Copy in *Silene latifolia*

## Supporting Information for Kazama *et al.*, 2012

**Files in this Data Supplement:**

- Supporting Information - Figures S1 and S2 and Tables S1-S3 (PDF, 928 KB)
- Figure S1 - Southern blot analysis of *SlWUS* genes under low stringency conditions (PDF, 307 KB)
- Figure S2 - An example of PCR analysis for detecting *SlWUS1* and *SlWUS2* orthologues in other dioecious species using degenerate primer sets (PDF, 656 KB)
- Table S1 - List of oligonucleotide primers used for sequencing *WUS* orthologues (PDF, 62 KB)
- Table S2 - List of oligonucleotide primers used in HRM (PDF, 51 KB)
- Table S3 - Result of QRT-PCR on inter-strain cross between the K-line male and a B female using allele specific primers of *SlWUS1* (PDF, 62 KB)
