# Supplementary material for: SlWUS1; An X-linked Gene Having No Homologous Y-Linked Copy in Silene latifolia
Source: G3 (Bethesda). 2012 Oct 1;2(10):1269–78. doi: 10.1534/g3.112.003749 (PMC3464119; doi:10.1534/g3.112.003749)
Supplement: Supporting Information [file supp_2.10.1269_003749SI.pdf]

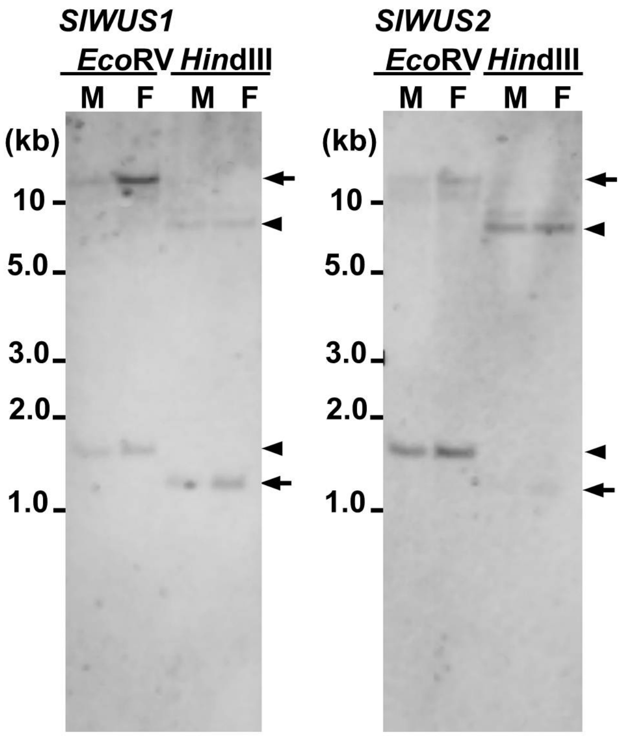

Figure S1 Southern blot analysis of *SlWUS* genes under low stringency conditions. Male and female genomic DNAs digested with *Eco*RV and *Hin*dIII were hybridized with probes that are complementary to the highly conserved homeodomain regions under low stringency conditions (See Materials and Methods). Both probes cross-hybridized to the other copies. Arrows and arrow heads indicate signals for *SlWUS1* and *SlWUS2*, respectively.

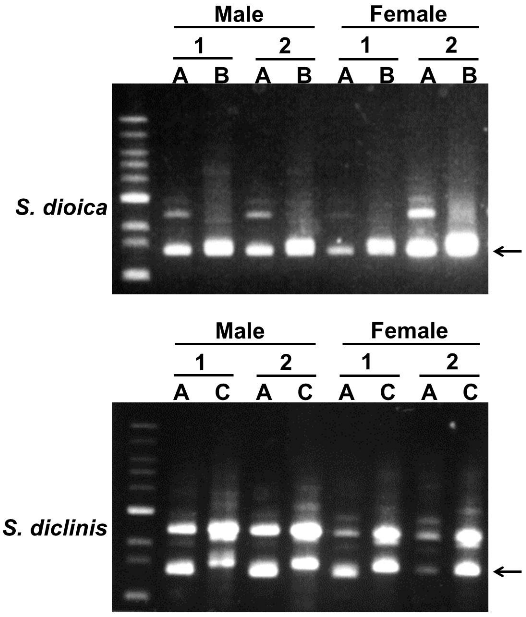

Figure S2 An example of PCR analysis for detecting *SlWUS1* and *SlWUS2* orthologues in other dioecious species using degenerate primer sets. Genomic DNAs isolated from Individual two male and female plants were used. A, B and C indicates the name of primer sets (A, kWUS5f: 5′-TGG TTY CAR AAY CAT AAA GC-3′ and kWUS2r: 5′-TCY BCR TGC ATN GGR AAT AG-3′; B, kWUS6f: 5′- TGG TTY CAR AAY CAC AAA GC-3′ and kWUS2r; C, kWUS7f: 5′- TGG TTY CAR AAY CAT AAG GC-3′ and kWUS2r). Several fragments were amplified in some PCRs and all fragments were cloned and sequenced. As a result, 1.6 kb and 1.7 kb fragments (indicated by an arrow in each panel) were ideintified as *SlWUS1* and *SlWUS2* orthologues, respectively. None of Y-specific fragment was detected.

Table S1 List of oligonucleotide primers used for sequencing *WUS* orthologues.

Primer name	Sequence (5’-3’)
SlWUS1F8	ATC TCA ACA AGT CCT TCT CTT GCA G
SlWUS1F9	CCC CCA CAT TAC CAT AAC TCC CTC T
SlWUS1R6	TTG GGG AGG ATC AAG TCT TTT GCT T
SlWUS1R7	TAA GAA AAC GAC TCC CCT ATA CGG A
dicWUS1R1	TGT CTC TAT CTC GGA TAC TGC AAC A
dioWUS_SR1	CTG GAG GTG GGC AAT TAG TAG GGA
SlWUS1_r1	TAT GCG GTG ACC ACT GCA TCA A
SlWUS1_sR3	ATG CTT GTT CTT GGC TCT TCA TT
SlWUS1_sR4	ACT CCA ATT TAG GTG ATT TAC TGG G
SlWUS2_F9	TCA GTT TCA TCC TTC ATT CCT TCC A
SlWUS2_F10	AAA TGG AAG GAC AAC CAA ACC AAC T
SlWUS2_R4	CAT CAA CGG GTC TTG TTC CTC TCT T
SlWUS2_R5	CCA AGT AGA GAA TTA GAG AGA ACC T
SlWUS2_f1	AGG CTA GGG AGA GAC AGA AG
SlWUS2_r1	CTC TAT CTC TGG CTG TGC TA
SlWUS2_sF3	CTT ACA GTA TTA AGA CAG CAA CAC C
SlWUS2_sF4	ACT AAT CAT TTA TTC GGT GGG TCT T

Table S2 List of oligonucleotide primers used in HRM.

Gene	Primer name	Sequence (5’-3’)
DD44X	DD44XhF	CCC TGC CCG AGA ATT TCC TG
	DD44XhR	TGG AAG GCT GAG GCA TGT GG
SlWUS1	SlWUS1hF	TGC AAT GAA AGG GGG CAA AG
	SlWUS1hR	TGA AGA GCC AAG AAC AAG CAT
SlX1	SlX1hF	AGT GGA GTT GGA TCA CCT GTT C
	SlX1hR	TAT GCG GTG ACC ACT GCA TCA A
SlX4	SlX4hF	CAA GAA TGC TGC AAT ACA GTC A
	SlX4hR	CTG GAT CTA CTT CAG AGA CAC C

Table S3 Result of QRT-PCR on inter-strain cross between the K-line male and a B female using allele specific primers of *SlWUS1*.

Samples	Relative expression level (K/B)	Standard deviation
F1-1	1.13	0.07
F1-2	1.09	0.19
F1-3	0.872	0.21

Relative expression level in K-line and B-line could not be calculated, because they expressed only one individual allele.
