# Supplementary material for: SlWUS1; An X-linked Gene Having No Homologous Y-Linked Copy in Silene latifolia
Source: G3 (Bethesda). 2012 Oct 1;2(10):1269–78. doi: 10.1534/g3.112.003749 (PMC3464119; doi:10.1534/g3.112.003749)
Supplement: Supporting Information [file supp_2.10.1269_FigureS1.pdf]

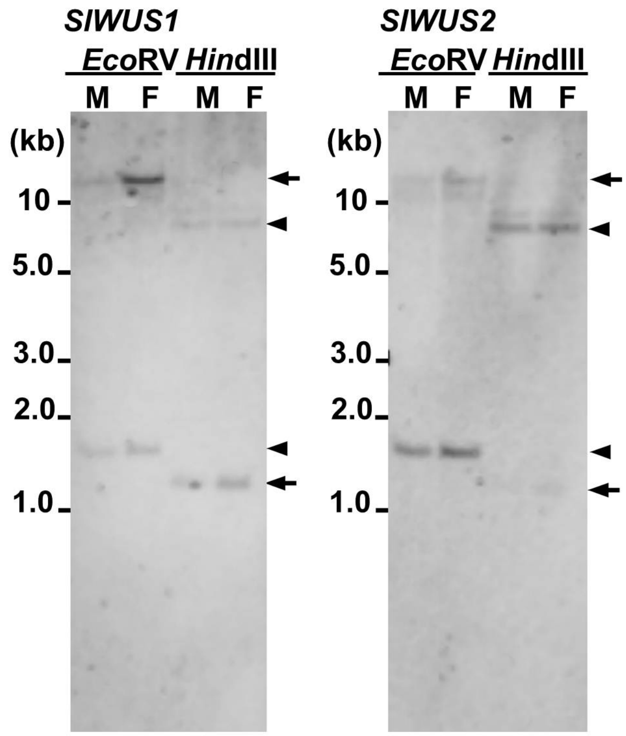

Figure S1 Southern blot analysis of *SlWUS* genes under low stringency conditions. Male and female genomic DNAs digested with *Eco*RV and *Hin*dIII were hybridized with probes that are complementary to the highly conserved homeodomain regions under low stringency conditions (See Materials and Methods). Both probes cross-hybridized to the other copies. Arrows and arrow heads indicate signals for *SlWUS1* and *SlWUS2*, respectively.
