# Supplementary material for: SlWUS1; An X-linked Gene Having No Homologous Y-Linked Copy in Silene latifolia
Source: G3 (Bethesda). 2012 Oct 1;2(10):1269–78. doi: 10.1534/g3.112.003749 (PMC3464119; doi:10.1534/g3.112.003749)
Supplement: Supporting Information [file supp_2.10.1269_FigureS2.pdf]

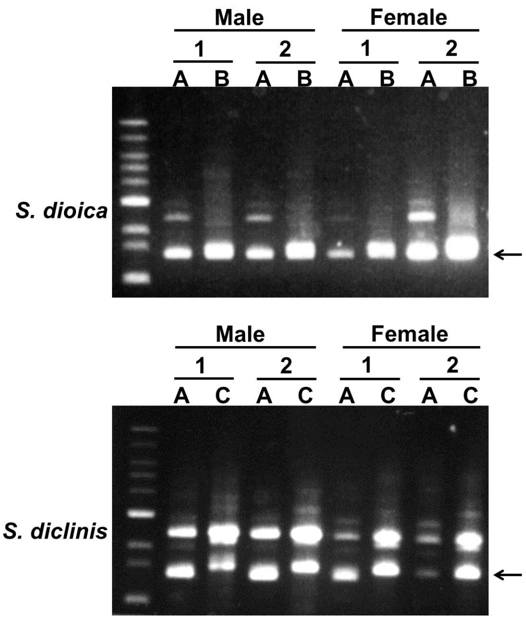

Figure S2 An example of PCR analysis for detecting *SlWUS1* and *SlWUS2* orthologues in other dioecious species using degenerate primer sets. Genomic DNAs isolated from Individual two male and female plants were used. A, B and C indicates the name of primer sets (A, kWUS5f: 5′-TGG TTY CAR AAY CAT AAA GC-3′ and kWUS2r: 5′-TCY BCR TGC ATN GGR AAT AG-3′; B, kWUS6f: 5′- TGG TTY CAR AAY CAC AAA GC-3′ and kWUS2r; C, kWUS7f: 5′- TGG TTY CAR AAY CAT AAG GC-3′ and kWUS2r). Several fragments were amplified in some PCRs and all fragments were cloned and sequenced. As a result, 1.6 kb and 1.7 kb fragments (indicated by an arrow in each panel) were ideintified as *SlWUS1* and *SlWUS2* orthologues, respectively. None of Y-specific fragment was detected.
