# Supplementary material for: SlWUS1; An X-linked Gene Having No Homologous Y-Linked Copy in Silene latifolia
Source: G3 (Bethesda). 2012 Oct 1;2(10):1269–78. doi: 10.1534/g3.112.003749 (PMC3464119; doi:10.1534/g3.112.003749)
Supplement: Supporting Information [file supp_2.10.1269_TableS1.pdf]

Table S1 List of oligonucleotide primers used for sequencing *WUS* orthologues.

Primer name	Sequence (5'-3')
SlWUS1F8	ATC TCA ACA AGT CCT TCT CTT GCA G
SlWUS1F9	CCC CCA CAT TAC CAT AAC TCC CTC T
SlWUS1R6	TTG GGG AGG ATC AAG TCT TTT GCT T
SlWUS1R7	TAA GAA AAC GAC TCC CCT ATA CGG A
dicWUS1R1	TGT CTC TAT CTC GGA TAC TGC AAC A
dioWUS_SR1	CTG GAG GTG GGC AAT TAG TAG GGA
SlWUS1_r1	TAT GCG GTG ACC ACT GCA TCA A
SlWUS1_sR3	ATG CTT GTT CTT GGC TCT TCA TT
SlWUS1_sR4	ACT CCA ATT TAG GTG ATT TAC TGG G
SlWUS2_F9	TCA GTT TCA TCC TTC ATT CCT TCC A
SlWUS2_F10	AAA TGG AAG GAC AAC CAA ACC AAC T
SlWUS2_R4	CAT CAA CGG GTC TTG TTC CTC TCT T
SlWUS2_R5	CCA AGT AGA GAA TTA GAG AGA ACC T
SlWUS2_f1	AGG CTA GGG AGA GAC AGA AG
SlWUS2_r1	CTC TAT CTC TGG CTG TGC TA
SlWUS2_sF3	CTT ACA GTA TTA AGA CAG CAA CAC C
SlWUS2_sF4	ACT AAT CAT TTA TTC GGT GGG TCT T
