# Supplementary material for: SlWUS1; An X-linked Gene Having No Homologous Y-Linked Copy in Silene latifolia
Source: G3 (Bethesda). 2012 Oct 1;2(10):1269–78. doi: 10.1534/g3.112.003749 (PMC3464119; doi:10.1534/g3.112.003749)
Supplement: Supporting Information [file supp_2.10.1269_TableS2.pdf]

Table S2 List of oligonucleotide primers used in HRM.

Gene	Primer name	Sequence (5'-3')
DD44X	DD44XhF	CCC TGC CCG AGA ATT TCC TG
	DD44XhR	TGG AAG GCT GAG GCA TGT GG
SlWUS1	SlWUS1hF	TGC AAT GAA AGG GGG CAA AG
	SlWUS1hR	TGA AGA GCC AAG AAC AAG CAT
SlX1	SlX1hF	AGT GGA GTT GGA TCA CCT GTT C
	SlX1hR	TAT GCG GTG ACC ACT GCA TCA A
SlX4	SlX4hF	CAA GAA TGC TGC AAT ACA GTC A
	SlX4hR	CTG GAT CTA CTT CAG AGA CAC C
