# Supplementary material for: SlWUS1; An X-linked Gene Having No Homologous Y-Linked Copy in Silene latifolia
Source: G3 (Bethesda). 2012 Oct 1;2(10):1269–78. doi: 10.1534/g3.112.003749 (PMC3464119; doi:10.1534/g3.112.003749)
Supplement: Supporting Information [file supp_2.10.1269_TableS3.pdf]

Table S3 Result of QRT-PCR on inter-strain cross between the K-line male and a B female using allele specific primers of *SlWUS1*.

Samples	Relative expression level (K/B)	Standard deviation
F1-1	1.13	0.07
F1-2	1.09	0.19
F1-3	0.872	0.21

Relative expression level in K-line and B-line could not be calculated, because they expressed only one individual allele.
